# Management of a Spontaneous Secondary Pneumothorax Complicated by Persistent Air Leak With Autologous Blood Patch Pleurodesis: A Case Report

**DOI:** 10.1155/crpu/6395001

**Published:** 2026-11-10

**Authors:** Ashok Basnet, Bhushan Kumar Rajbhandari, Ekta Malla Rana, Rajesh K. C., Saroj Raj K. C., Anu Bohara, Bhupesh Chataut

**Affiliations:** ^1^ Department of Pulmonary Medicine Shree Birendra Hospital Kathmandu Nepal; ^2^ Department of Radiodiagnosis and Imaging Shree Birendra Hospital Kathmandu Nepal; ^3^ Department of Internal Medicine Nepalese Army Institute of Health Sciences Kathmandu Nepal naihs.edu.np

**Keywords:** autologous blood patch pleurodesis, effective, low-cost, persistent air leak, safe

## Abstract

Persistent air leak (PAL) in pneumothoraces, especially in underlying structural lung disease, remains a significant challenge leading to prolonged hospitalization and even mortality because of pleural sepsis. Various treatment modalities, such as chemical pleurodesis, autologous blood patch, endobronchial valve, and surgical interventions, have been reported in the literature. We report a case of an 80‐year‐old male with PAL successfully managed with 100 mL of autologous blood patch pleurodesis (ABPP) as the patient was deemed unsuitable for surgery. In our experience, ABPP has proven to be safe, bedside, low‐cost effective procedure with minimal complications. ABPP is still underutilized and is used mostly in resource‐limited settings. We believe ABPP should be accepted as a treatment option before considering surgery in selected patients.

## 1. Introduction

Spontaneous pneumothorax, in structural lung diseases such as emphysema and bullous lung disease, can be complicated by air leaks, accounting for up to 30% [1]. Air leak is considered persistent when present for more than 5–7 days. Despite lung expansion post chest tube insertion, persistent air leak (PAL) remains a challenge, prolonging the hospital duration with increase in risk of pleural infection. When the patient is not fit for surgical intervention and the availability of endobronchial interventions is limited, the remaining therapeutic options are chemical pleurodesis and autologous blood patch pleurodesis (ABPP) [2, 3]. Considering air leak, ABPP, which is safe and cost‐effective, remains the therapeutic choice [4]. We present a case of secondary pneumothorax with PAL managed successfully with ABPP.

## 2. Case Presentation

An 80‐year‐old man, with no known prior comorbidities, current daily cigarette smoker with 70 pack‐years, presented to the emergency department with acute‐onset right‐sided chest pain and shortness of breath of 2 days duration. At presentation, he was tachypneic, with a respiratory rate of 36/min, a resting oxygen saturation of 82% at room air, a heart rate of 110 beats/min, and a blood pressure of 150/90 mm Hg. On examination of right hemithorax, percussion was hyperresonant and breath sounds were absent. On left hemithorax, expiratory wheeze was heard. His chest radiograph revealed a large right‐sided pneumothorax (Figure 1a), and arterial blood gas analysis showed moderate hypoxemia with Type I respiratory failure. Subsequently, a 24F intercostal chest drain was inserted in the right hemithorax and connected to water seal drainage, which showed bubbling of air during expiration only (Cerfolio Grade 2) (Table 1). His HRCT thorax showed residual small pneumothorax (Figure 2a), subcutaneous emphysema, along with significant bilateral emphysematous lungs, and multiple bullae (Figure 2b). After 10 days of chest drainage, air leak persisted. Thoracic surgeon opinion was taken for consideration of surgical intervention (VATS). He had risk factors such as advanced age, baseline functional status (ECOG PS‐3), underweight (BMI: 17.26 kg/m^2^), poor nutritional status (serum albumin: 2.3 g/dL, Hb: 10.8 g/dL), significant bilateral emphysema with multiple bullae and severe pulmonary hypertension (ePASP: 65 mm Hg). Considering these risk factors, he was deemed unfit for thoracic surgery. In a resource‐limited setting, pleurodesis was the only viable medical management option therefore, we proceeded with ABPP.

**Figure 1:**
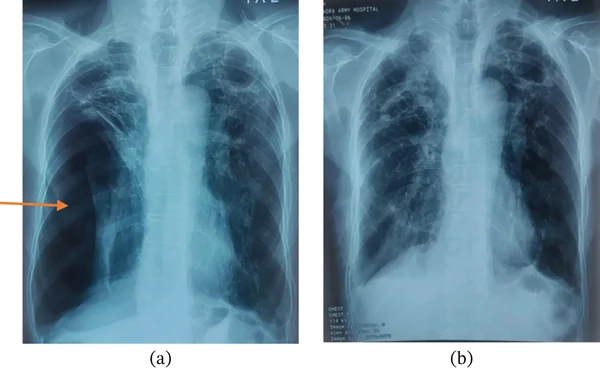
(a) Pre‐ABPP chest radiograph showing large right‐sided pneumothorax (red arrow). (b) Post‐ABPP chest radiograph showing resolution of right‐sided pneumothorax.

**Table 1:** Cerfolio classification of air leaks.

Grade 1	Air bubbles appear during forced expiration or while coughing
Grade 2	Expiration only
Grade 3	Inspiration only
Grade 4	Continuous bubbling present during inspiration and expiration

*Note:* Adapted from *Chest*, 152(2), 417‐423, by Dugan et al., 2017.

**Figure 2:**
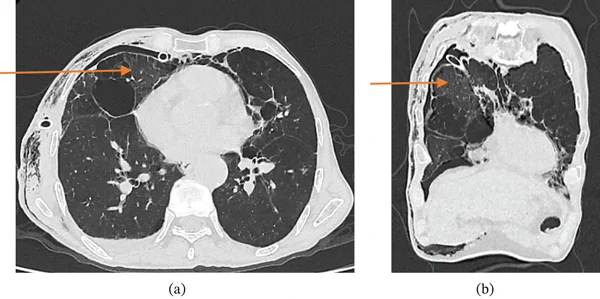
(a) Representative CT thorax axial image showing residual right‐sided pneumothorax (red arrow). (b) Representative CT thorax coronal image showing surgical emphysema, emphysematous lungs, and bullae with in situ chest tube (red arrow).

## 3. Procedure

The patient was placed in lateral position with the left side down. Using a 2 mL/kg protocol, unheparinized blood was drawn from the left antecubital vein with 20‐mL sterile syringe (five aliquots) and rapidly instilled through a three‐way stopcock into the elevated chest tube; total blood volume 100 mL (body weight 48 kg) was instilled. After each blood instillation, the tube was flushed aseptically with 10 mL normal saline (total volume 50 mL) to prevent clotting (Figure 1). No suction was applied, and the chest tube was not clamped because of tension pneumothorax risk. The tube was then connected to a water seal drain, elevated above the thorax, and secured to an IV stand for 2 hours to prevent blood drainage. The patient was repositioned every 15 minutes during the first hour (lateral → supine → Trendelenburg) to distribute blood evenly and coat the pleura. We believed repositioning technique would seal apical air leaks. Because the air leak persisted, a second ABPP session was performed 72 hours later using the same protocol. The air leak ceased completely 48 hours after the second session.

The chest radiograph showed resolution of pneumothorax (Figure 1b), then the drain was removed. He was discharged after weaning oxygen support. His repeat HRCT thorax showed complete resolution of right‐sided pneumothorax (Figure 3a) with apical fibrosis (Figure 3b) suggesting no recurrence during the 4‐week follow‐up period.

**Figure 3:**
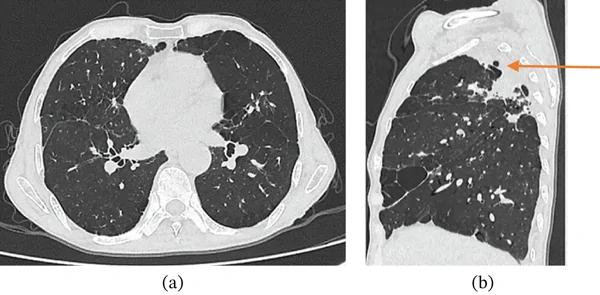
(a) Follow‐up CT thorax axial image showing complete resolution of right‐sided pneumothorax. (b) Follow‐up CT thorax sagittal image showing apical fibrosis (red arrow).

## 4. Discussion

Talc chemical pleurodesis can be highly effective compared with other agents as shown by Cobanoglu et al. study [5], but chemical pleurodesis can be associated with adverse effects in PAL, so in our patient we decided to proceed with ABPP. Robinson [6] in 1987 first discussed successful autologous blood pleurodesis. Since then, ABPP has found its space in both post–lung surgery and spontaneous pneumothoraces, with a success rate up to 92% [7]. There is no consensus on the exact blood volume. Various studies have used blood volume from 50 mL up to 200 mL [8]. We instilled 100 mL blood.

Instilled blood forms a fibrin clot directly over the site of the air leak, creating a physical meshwork that reduces or stops the leak and allows the lung to re‐expand. It also stimulates local inflammation by releasing cytokines and growth factors, recruiting fibroblasts, and promoting collagen deposition, ultimately leading to permanent pleural adhesion [1, 9]. In a study by Cobanoglu et al. [5], ABPP has shown effectiveness up to 75%, compared with other pleurodesis agents. It should be considered in selected spontaneous pneumothorax patients who are high‐risk candidates for surgical intervention as per conditional recommendation by the 2024 Joint ERS/EACTS/ESTS clinical practice guideline [10] and post–lung resection prolonged air leaks [11]. Some procedure‐related side effects, like fever and empyema, have been mentioned in the literature [1]; however, when a strict aseptic protocol is followed, the incidence of complications is less than 10% [12]. In our patient, no such side effects were seen. Contraindication to ABPP (Table 2), Pathak et al. [13] mentioned that the procedure can be repeated as needed, as in our case, air leak ceased 48 hours following the second session of blood patch.

**Table 2:** Contraindication to ABPP.

Absolute contraindication	Relative contraindication
Sepsis or septic shock	Large air leaks (drain flow > 300 mL/min)
	Smaller chest drain < 12F
Pleural empyema	Requirement for suction on the chest drain > 5 cm H_2_O
	Nonexpandable lung

*Note:* Adapted from *Pleural Disease*, Dansk Lungemedicinsk Selskab (DLS), by Fjællegaard et al., 2025.

ABPP is a simple, low‐cost, bedside procedure with few side effects and a high success rate for PAL after lung surgery, trauma, or spontaneous pneumothorax and even in not fully expanded nonentrapped lung [14], yet it remains underutilized. ABPP may be considered as a minimally invasive therapeutic option in selected patients with PAL, particularly those who are unsuitable for surgery [1, 13]. A 2023 systematic review and meta‐analysis published in Cureus on the efficacy and safety of autologous blood patch (ABP) [15] supports its use in patients with PALs, which is relevant to our complicated case. Although ABPP is not a novel procedure, it is considered when there are no options available. The main advantage is that it can be repeated multiple times, making it particularly valuable in elderly patients and resource‐limited settings. Although a large multicenter trial is unlikely, if one were conducted, it could provide a clearer understanding of how ABPP fits into the treatment paradigm for complicated patients with spontaneous pneumothorax and PAL.

## 5. Conclusion

ABPP is a minimally invasive, inexpensive, effective bedside procedure with minimal complications. It should be utilized as therapeutic option in selected patients not amenable to surgical intervention.

## Funding

No funding was received for this manuscript.

## Ethics Statement

Informed written consent was obtained from the patient for the publication of the report and all the procedures were performed in accordance with the Declaration of the Helsinki.

## Consent

Written informed consent was obtained from the patient for the publication of his clinical details and any accompanying images.

## Conflicts of Interest

The authors declare no conflicts of interest.

## Supporting information


**Supporting Information** Additional supporting information can be found online in the Supporting Information section. Figure S1: Autologous blood patch pleurodesis procedure: unheparinized blood withdrawn from antecubital vein and instilled via elevated chest drainage tube with normal saline flush.

## Data Availability

The data that supports the findings of this study are available in the supplementary material of this article.
