# Supplementary material for: Management of a Spontaneous Secondary Pneumothorax Complicated by Persistent Air Leak With Autologous Blood Patch Pleurodesis: A Case Report
Source: Case Rep Pulmonol. 2026 Nov 10;2026:6395001. doi: 10.1155/crpu/6395001 (PMC13749074; doi:10.1155/crpu/6395001)
Supplement: Supplementary file 1 [file CRPU-2026-6395001-s001.docx]

Fig S1- Image showing Autologous Blood patch procedure
